# Supplementary material for: The causal role of circulating inflammatory markers in osteoporosis: a bidirectional Mendelian randomized study
Source: Front Immunol. 2024 Jul 18;15:1412298. doi: 10.3389/fimmu.2024.1412298 (PMC11291241; doi:10.3389/fimmu.2024.1412298)
Supplement: Supplementary file 1 [file DataSheet_1.zip › supplementary material/OSTEOPOROSIS-CIM/GCST90274819.tsv.gz_leave_one_out.pdf]

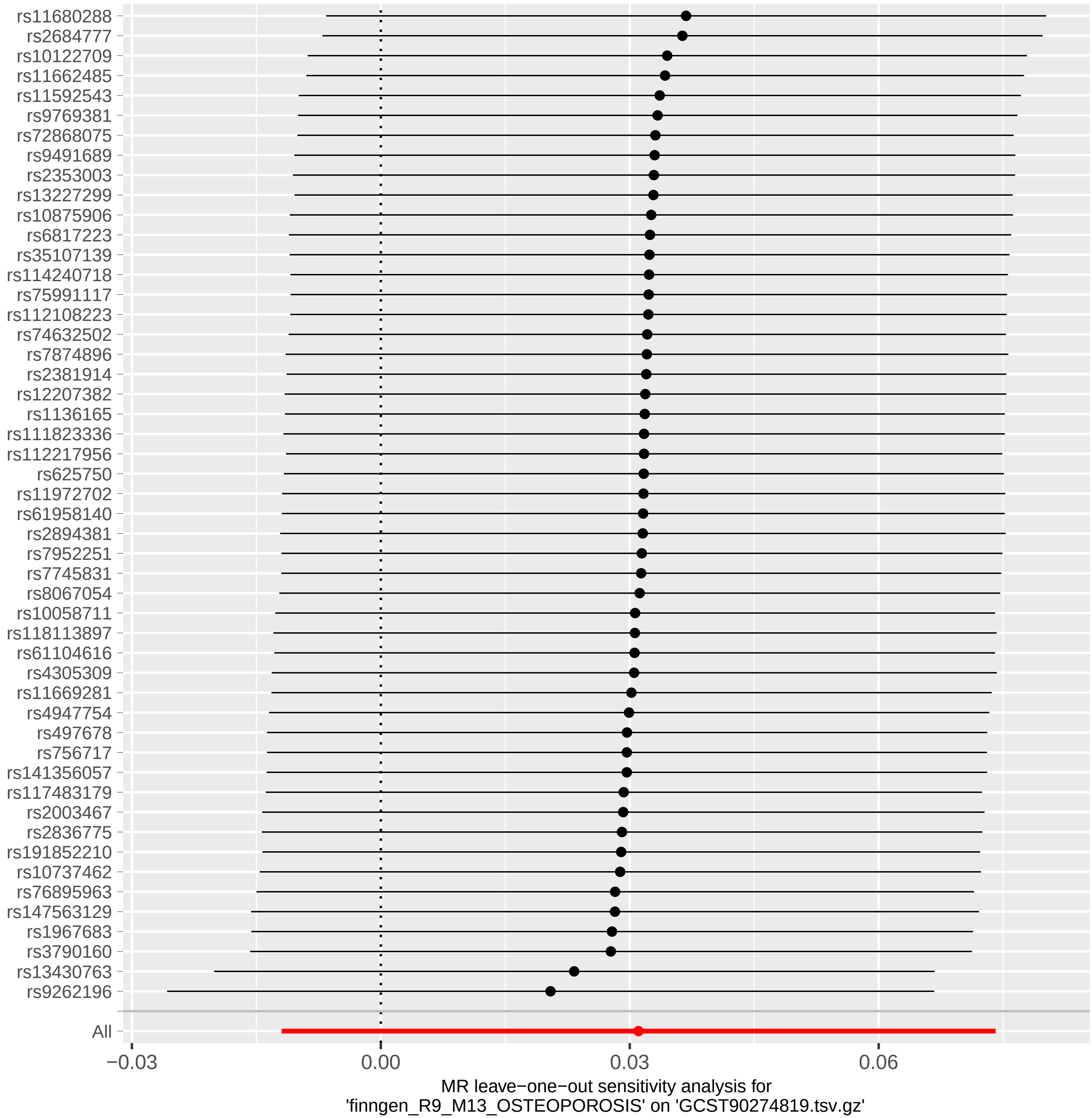
rs11680288
rs2684777
rs10122709
rs11662485
rs11592543
rs9769381
rs72868075
rs9491689
rs2353003
rs13227299
rs10875906
rs6817223
rs35107139
rs114240718
rs75991117
rs112108223
rs74632502
rs7874896
rs2381914
rs12207382
rs1136165
rs111823336
rs112217956
rs625750
rs11972702
rs61958140
rs2894381
rs7952251
rs7745831
rs8067054
rs10058711
rs118113897
rs61104616
rs4305309
rs11669281
rs4947754
rs497678
rs756717
rs141356057
rs117483179
rs2003467
rs2836775
rs191852210
rs10737462
rs76895963
rs147563129
rs1967683
rs3790160
rs13430763
rs9262196
All
−0.03
0.00
0.03
0.06
MR leave−one−out sensitivity analysis for
'finngen_R9_M13_OSTEOPOROSIS' on 'GCST90274819.tsv.gz'
